# Prehabilitation in an Integrative Medicine Day Clinic for Patients Undergoing Neoadjuvant Treatment: Single-Center Feasibility Pilot Study

**DOI:** 10.2196/46765

**Published:** 2023-10-18

**Authors:** Christian Raff, Colette Dörr-Harim, Stephanie Otto, Johanna Thiele, Andre Mihaljevic, Klaus Kramer

**Affiliations:** 1 Department of General and Visceral Surgery Working Group of Integrative Medicine University Hospital Ulm Ulm Germany; 2 Department of General, Visceral and Transplantation Surgery Heidelberg University Hospital Heidelberg Germany; 3 Comprehensive Cancer Center Ulm University Hospital Ulm Ulm Germany; 4 Department of General, Visceral and Transplantation Surgery University Hospital Tübingen Tübingen Germany

**Keywords:** supportive care, prehabilitation, neoadjuvant treatment, integrative medicine, multimodal prehabilitation, cancer, oncology, surgery, preoperative, feasibility, integrative, naturopathy, naturopathic, diet, nutrition

## Abstract

**Background:**

Patients with cancer receiving neoadjuvant treatment prior to surgery are in a very stressful situation. Chemotherapy and radiation therapy put a strain on the quality of life and the pending surgery poses a relevant burden for many patients. Preparation of these patients for the intervention in terms of prehabilitation has great potential to reduce the burden of postoperative complications and may improve the clinical outcome. A prehabilitation approach also yields the possibility to address unmet patients’ needs and to help them modify their lifestyle in a maintainable way. Therefore, a multimodal approach is mandatory during this critical period.

**Objective:**

The aim of this study is to assess the feasibility of prehabilitation in an integrative medicine day clinic (PRIME-DC) prior to cancer surgery at a major university clinic. PRIME-DC is considered feasible if 80% of enrolled patients are willing and able to complete at least 6 out of the 8 weekly meetings, each lasting 6.5 hours, at such a clinic. Secondary end points aim to evaluate this multimodal program.

**Methods:**

The PRIME-DC intervention combines mind-body medicine, exercise therapy, nutrition therapy, naturopathic counseling, and the application of a yarrow liver compress. Adult patients with cancer, with a primary tumor in the abdomen (including intraperitoneal cancer, stomach cancer, and extraperitoneal cancers such as pancreatic, bladder, rectal, esophageal, endometrial, ovarian, and cervical cancer) or the breast requiring a neoadjuvant oncological treatment setting are eligible to participate. The addressed cancer entities imply either an extensive surgical intervention with an expected need for prehabilitation (eg, abdominal surgery) or a neoadjuvant treatment of several months with a high burden of treatment-associated side effects (breast cancer). Adherence to the day clinic program is the primary end point being defined as presence during the day clinic session. Secondary end points are physical assessment and quality of life, together with a structured assessment of neoadjuvant treatment-associated side effects. Furthermore, to collect qualitative data voluntary participants of the day clinic will be interviewed in a semistructured way after completion of the day clinic program on each component of the study (mind-body intervention, exercise, nutrition, naturopathic counseling, and a yarrow liver compress).

**Results:**

The procedures used in this study adhere to the tenets of the Declaration of Helsinki. As of February 2023, we enrolled 23 patients; the dominant cancer entity is breast cancer (18 enrolled patients).

**Conclusions:**

The presented protocol combines prehabilitation, lifestyle modification, naturopathic counseling, dietary assistance, and naturopathic treatment in an innovative and integrative way.

**Trial Registration:**

Deutsches Register Klinischer Studien German Clinical Trials Register DRKS00028126; https://drks.de/search/de/trial/DRKS00028126

**International Registered Report Identifier (IRRID):**

DERR1-10.2196/46765

## Introduction

Surgical interventions are burdensome for patients with cancer. Especially the period between diagnosis and surgery is a major challenge for the patient in many ways. Besides the psychological strain of the upcoming intervention, the physiological burden of an extensive surgery, for example, an abdominal operation can be compared with running a marathon [1]. The more extensive the surgery, the higher the rate of relevant postoperative complications, particularly in patients who are frail and those with an extensive medical history.

Conventional treatment plans include rehabilitation after medical or surgical treatment. Innovative concepts developed in the mid-1990s use the preoperative phase for various measures to improve patients’ postoperative results with fewer and less severe complications. Analysis of these concepts of “prehabilitation” indicated efficacy [2,3]. The initial results were positive even in rather short (2-4 weeks) interventions comprising several sessions.

However, there is growing evidence that these early prehabilitation concepts lacked fundamental aspects of preparation [4]. First, they did not address anxiety, patients’ empowerment, or strengthening of self-efficacy, which is well known to have a beneficial effect on cancer care [5]. Moreover, the idea arose that preparation for extended surgery represents a “teachable moment,” a point in the life of a patient where long-lasting lifestyle modification is possible [6].

To date, no such program has been installed at the University Hospital Ulm. Nonetheless, the period of neoadjuvant chemotherapy, extending over a number of months provides time for a concise, integrative prehabilitation program addressing more than just the physical aspect of prehabilitation.

A promising integrative therapy for lifestyle modification, stress relief, and reduction of anxiety is mind-body medicine (MBM). The National Cancer Institute defines mind-body practice as “a health practice that combines mental focus, controlled breathing, and body movements to help relax the body and mind. It may be useful to control pain, stress, anxiety, and depression, and for overall health” [7]. The combination of mind-body practice, which includes a variety of mindfulness-based interventions, with aspects of cognitive behavioral therapy to enhance stress-coping mechanisms, is called MBM. This integrative treatment modality was established and adapted for a day clinic setting by Paul et al [8] and Dobos and Paul [9]. The concept of MBM within a day-hospital setting is traditionally applied to groups of 8-12 patients and is believed to be most effective in this group size with respect to lifestyle modification and stress management.

MBM shows positive effects in the treatment of fatigue [10] in patients with cancer while reducing anxiety about cancer recurrence [11]. Assuming the existence of a rationale for applying MBM in advance of surgery, it is noteworthy that postoperative pain and use of opioids are reduced even with a short MBM intervention, as shown by Pester et al [12] and Hanley et al [13]. The group setting is a hallmark of MBM therapy and enables the patients to learn from one another.

Another approach to enhance patients’ self-efficacy and address an unmet need in the treatment of patients with cancer may be the integration of naturopathic therapy in an evidence-based manner. There is evidence that naturopathic counseling enhances quality of Life (QoL) of patients with cancer [14]. Many patients use naturopathic methods as an additional complementary therapy, commonly without informing their oncologists [15] and without being aware of the risks of interactions between naturopathic medication and their chemotherapy. In order to save patients from falling for false claims or risking adverse effects, the Association of the Scientific Medical Societies in Germany has issued a medical guideline for the complementary treatment of patients with cancer [16]. The evidence-based foundation of this guideline enables empowering counseling of patients with cancer about how to deal with cancer- or treatment-related side effects. Work on the establishment of a structured counseling program based on this guideline is in progress [17].

The yarrow liver compress is included in the day clinic setting as it has been shown to be effective against treatment-associated fatigue [18] and to increase sympathetic modulation assessed by heart-rate variability [19]. The main topic of this session is to give the patients an experience of a specific naturopathic treatment that is recommendable for fatigue and yields a very low risk of adverse effects.

The combination of harmonizing supportive therapies might meet the needs of highly vulnerable patients during neoadjuvant treatment facing surgery. None of the named interventions has been used to support prehabilitation at University Hospital Ulm so far.

Preoperative multimodal interventions such as exercise, nutrition, and mindfulness-based treatments are thought to improve patients’ physical, nutritional, and mental status. Furthermore, cancer prehabilitation may improve patients’ participation in rehabilitation after cancer treatment and maintain their ability to engage in salutogenic activities. Such an integrative approach should include exercise for physical prehabilitation, mindfulness-based interventions to improve patients’ ability to cope with stress and anxiety prior to surgery, cognitive behavioral aspects to change high-risk lifestyles for the better, and last but not least, empowerment of patients to apply naturopathic therapies independently in an evidence-based manner. This combination of measures represents a promising portfolio and may show strong synergistic effects.

## Methods

### Overview

This study is a single-center, single-arm interventional study evaluating the feasibility of an 8-week prehabilitation program before, during, or after neoadjuvant treatment before surgery at University Hospital Ulm.

### Recruitment

During the recruitment period, all patients who are being treated for abdominal or breast cancer at the University Hospital Ulm on the Comprehensive Cancer Center Ulm and for whom neoadjuvant treatment is planned or performed with curative intent are screened for eligibility. Ineligible patients are listed, and in the case of refusal to participate, the reason, if communicated, is documented. Up to 15 patients who have given informed consent are enrolled in each cycle of the study (see sample size calculation). The addressed cancer entities imply either an extensive surgical intervention with an expected need for prehabilitation (eg, abdominal surgery) or a neoadjuvant treatment of several months with a high burden of treatment-associated side effects (breast cancer).

Patients may participate in the pilot study if they meet all of the following criteria: (1) diagnosis with intracavity cancer (including stomach cancer and extraperitoneal tumors such as prostate, bladder, rectal, esophageal, endometrial, and cervical cancer) or breast cancer; (2) planned or ongoing neoadjuvant treatment prior to planned curative resection; (3) aged 18 years or older; and (4) ability to understand the nature and individual consequences of the clinical trial. Patients are excluded from participation if their American Society of Anesthesiologists physical status classification system grade is >3, if they are immobile or unable to walk unassisted, or if they are already enrolled in a study that would interfere with the intervention and outcome of this trial.

### Intervention

#### Overview

The intervention consists of a standardized MBM program (a group-based semi-individual approach to address lifestyle medicine and to enhance mindfulness) and naturopathic counseling, which is both led by a trained doctor and mind-body therapist, application of a yarrow liver compress which is applied by specially trained nurses, physical training therapy which is led by a sports therapist, a 1-day intervention in a teaching kitchen, and an organic lunch provided by a local caterer comprising a salad, a vegetarian protein-rich meal, and a dessert. The day clinic is scheduled once weekly, each session lasting 6.5 hours. This duration is common among other day clinics in the same setting [8]. The approach is group-based to enhance social support during the process. A detailed plan of the sessions and the scheduled topics is presented in Table 1.

Apart from allergic reactions to yarrow, no specific adverse effects are expected. A casualty insurance is contracted.

The study protocol has been presented to and discussed with patient representatives, the heads of the department of gynecology, urology, gastro-oncology, and radiation therapy, and the director of Comprehensive Cancer Center Ulm. This steering committee is informed intermittently about the status of the study.

**Table 1:** The weekly schedule of day clinic activities.

Week	Activity (duration)
1	Questionnaire (15 min) Get together or group forming (75 min) Exercise therapy (60 min) Lunch (45 min) Yarrow liver compress (60 min) Introduction to mind-body-medicine (105 min) Meditation (30 min)
2	Questionnaire (15 min) Week review (30 min) Naturopathic consult (45 min) Exercise therapy (60 min) Lunch (45 min) Yarrow liver compress (60 min) Stress enhancing thought (105 min) Meditation (30 min)
3	Questionnaire (15 min) Week review (30 min) Naturopathic consult (45 min) Exercise therapy (60 min) Lunch (45 min) Yarrow liver compress (60 min) Reflection about personal stress response (105 min) Meditation (30 min)
4	Questionnaire (15 min) Group cooking (255 min) Yarrow liver compress (60 min) Week review and naturopathic consult (30 min) Meditation (30 min)
5	Questionnaire (15 min) Week review (30 min) Naturopathic consult (45 min) Exercise therapy (60 min) Lunch (45 min) Yarrow liver compress (60 min) Stress and communicating with oneself (105 min) Meditation (30 min)
6	Questionnaire (15 min) Week review (30 min) Naturopathic consult (45 min) Exercise therapy (60 min) Lunch (45 min) Yarrow liver compress (60 min) Changing habits (105 min) Meditation (30 min)
7	Questionnaire (15 min) Week review (30 min) Naturopathic consult (45 min) Exercise therapy (60 min) Lunch (45 min) Yarrow liver compress (60 min) Mindfulness retreat (105 min) Meditation (30 min)
8	Questionnaire (15 min) Week review (30 min) Naturopathic consult (45 min) Exercise therapy (60 min) Lunch (45 min) Yarrow liver compress (60 min) Review of day clinic and future plans (105 min) Meditation (30 min)

#### Mind-Body Intervention

We use a modified version of the standardized MBM approach established by Dobos and Paul [9]. The mind-body intervention combines aspects of the MBSR program developed by Kabat-Zinn and elements of the MBM cancer program established by Henry Benson at Harvard Medical School [20]. It combines relaxation techniques and group-based cognitive therapy to address lifestyle modification. Patients have access to digital copies of verbal instructions (script and audio files) and are encouraged to practice mindfulness techniques at home. The therapist in charge of the mind-body intervention is a board-certified internist and attended an advanced training as a mind-body-medicine therapist described in Mind-Body Medicine in Integrative and Complementary Medicine [9].

#### Naturopathic Counseling

Naturopathic counseling is known to enhance the QoL of patients with cancer [14] and a high percentage of patients use naturopathic methods sometimes without informing their oncologists [15]. Therefore, everyday clinic session includes naturopathic counseling based on the current guideline for complementary medicine in oncology. The counseling aims to enhance patients’ self-efficacy and enables them to address neoadjuvant treatment-associated side effects at home. The counseling embraces topics such as teas, compresses, the importance of sleep and sleep-enhancing rituals, as well as aspects of aromatherapy. Patients are free to utter their personal complaints during the last days or questions about possible side effects of the neoadjuvant treatment and are given advice based on the current guidelines for complementary medicine in oncology.

#### Exercise Therapy Interventions

Prehabilitation exercise therapy includes a combination of strength, cardiovascular or aerobic training, yoga, qigong, and stretching exercises. The program focuses on the educational aspect, giving advice on additional home-based exercises. The duration of the supervised intervention is 1 hour a week and the intended intensity is moderate.

#### Liver Compress

A yarrow liver compress is applied after the patient has eaten lunch at the day clinic. The liver compresses are prepared and applied by trained nurses. We infuse 1 tablespoon of yarrow tea with cut blossoms, stems, and leaves of common yarrow (*Achillea millefolium*) with 250 mL boiling water, letting it infuse until the tea takes on a yellow color (approximately 7 min) for each patient. We soak a cotton cloth in the infusion and then wring it out. The cloth is then fanned and gently draped over the patient’s right upper abdomen. A towel is wrapped around the patient’s abdomen and a hot-water bottle is placed over the compress. We cover the patient with a blanket followed by a rest for 30 minutes. The cotton cloth is then removed and the patient rests for another 30 minutes.

#### Nutrition and Teaching Kitchen

Nutrition is addressed during each day’s clinic session. It may be a topic during naturopathic counseling depending on the individual needs of the participants. Each day clinic session includes an organic lunch provided by a local caterer. The participants eat together, which enhances group cohesion. One visit to the day clinic is dedicated to a session in a teaching kitchen. Participants prepare a meal under the guidance of a trained nutritionist. Apart from the group-forming aspect of preparing and eating a meal together, this session includes counseling about nutrition during cancer and cancer therapy focusing on the individual needs of the participants. In order to support the muscle status of the participants, they are counseled to adhere to a protein-rich (1-1.5 g/kg/day) diet without specific restrictions concentrating on maintaining the pretreatment weight with respect to the individual needs of each participant.

### Outcomes

Each patient’s adherence to the day clinic program is the primary end point measured via the noted presence during the day clinic sessions. The secondary end points are patient-reported outcomes assessed by means of the European Organisation for Research and Treatment of Cancer Quality of Life Questionnaire C30 (EORTC QLQ-C30) [21], the Common Terminology Criteria for Adverse Events (PRO-CTCAE) [22], the Quality of Recovery-15 score (QoR-15) [23], the 4-item patient health questionnaire for anxiety and depression (PHQ-4) [24], the Pittsburgh Sleep Quality Index (PSQI) [25], the Five-Facet Mindfulness Questionnaire (FFMQ-D) [26], and fatigue (using a numeric rating scale) and clinician-reported outcome measures such as postoperative complications ≥3 according to Dindo and Clavien [27] within 30 days after the index operation, duration of postoperative hospital stay, readmission after initial discharge from the index operation within 30 days, 6-minute walking distance (6-MWD), hand grip strength (HGS), the result of the 1-minute sit-to-stand test (1-MSTS), the intensity of analgesic treatment according to the WHO pain ladder, and adherence to neoadjuvant treatment during the day clinic program. The physical assessment is conducted by study personal. The times of assessment of the end points are shown in Table 2.

**Table 2:** The visit and corresponding time of data collection.

Visit		1	2-9	10^a^	10^b^	11	12	13
Time		Screening	Weeks 1-8 of prehabilitation	Post prehabilitation	1-10 days before surgery^c^	POD^d^ 4	Day of discharge	POD^d^ 30
**Inclusion**								
	Informed consent	✓	N/A^e^	N/A	N/A	N/A	N/A	N/A
	Eligibility criteria	✓	N/A	N/A	N/A	N/A	N/A	N/A
**Assessments**								
	Demographics and baseline clinical data	✓	N/A	N/A	N/A	N/A	N/A	N/A
	BMI	✓	N/A	✓	N/A	N/A	N/A	✓
**Questionnaires**								
	EORTC QLQ-C30^f^	✓	N/A	✓	✓	✓	N/A	✓
	PRO-CTCAE^g^	✓	✓	N/A	N/A	N/A	N/A	N/A
	Fatigue scale	✓	✓	✓	✓	✓	✓	✓
	QoR-15 GE^h^	✓	N/A	N/A	N/A	✓	N/A	N/A
	PHQ-4^i^	✓	N/A	✓	✓	N/A	N/A	✓
	Pittsburgh Sleep quality Index	✓	✓^j^	✓	✓	N/A	N/A	✓
	Five-Facet Mindfulness Questionnaire	✓	N/A	✓	✓	N/A	N/A	✓
**Physical examination**								
	6-minute walking test	✓	N/A	✓	N/A	N/A	N/A	N/A
	Hand grip strength using a dynamometer	✓	N/A	✓	N/A	N/A	N/A	N/A
	1-minute sit to stand test	✓	N/A	✓	N/A	N/A	N/A	N/A
Assessment of postoperative complications		N/A	N/A	N/A	N/A	✓	✓	✓
Intensity of analgesic treatment		✓	N/A	N/A	N/A	✓	✓	✓
Readmission to hospital		N/A	N/A	N/A	N/A	N/A	N/A	✓
Reoperation		N/A	N/A	N/A	N/A	N/A	N/A	✓
Time of stay on intensive care unit		N/A	N/A	N/A	N/A	N/A	N/A	✓
Adherence to neoadjuvant treatment		N/A	✓	N/A	N/A	N/A	N/A	N/A
Evaluation day clinic satisfaction		N/A	N/A	N/A	N/A	N/A	N/A	✓

^a^Only if time between end of prehabilitation and surgery >14 days.

^b^Every 4 weeks during prehabilitation.

^c^Only if time between end of prehabilitation and surgery >14 days.

^d^POD: postoperative day.

^e^N/A: not applicable.

^f^EORTC QLQ-C30: European Organisation for Research and Treatment of Cancer Quality of Life Questionnaire C30.

^g^PRO-CTCAE: Common Terminology Criteria for Adverse Events.

^h^QoR-15: Quality of Recovery-15 score.

^i^PHQ-4: 4-item patient health questionnaire for anxiety and depression.

^j^Every 4 weeks during prehabilitation.

### Statistical Analysis

#### Sample Size Calculation

Prehabilitation in an integrative medicine day clinic (PRIME-DC) is a pilot study with an explorative approach and will be held in 3 cycles. Thus, no formal sample size calculation is needed. A group size of 6-12 patients per cycle was considered reasonable for evaluation of feasibility and acceptance of the prehabilitation day clinic setting. Patients who withdraw are not replaced, which is inherent for a closed group. Therefore, a sample of 18-45 patients is to be recruited. As the patients follow a group-based MBM program, the sample size has to fit the range appropriate for an MBM therapy group, that is, 6-15 patients.

#### Data Analysis Plan

The data analysis will give a comprehensive picture of the data through descriptive statistics and assess the precision and certainty of estimates using different CI widths.

First, descriptive statistics are used to summarize and describe the main features of the data set. This includes measures such as mean, median, SD, minimum, maximum, and percentiles. These statistics provide a clear and concise summary of the data's central tendencies and dispersion.

Further, the impact of different CI widths on the results and conclusions is of interest. CIs with 3 different widths, namely 75%, 85%, and 95%, are planned to be calculated and compared. The estimate becomes more precise as the CI narrows, albeit with reduced certainty regarding the capture of the true parameter [28].

Once the CIs are calculated, they will be interpreted in the context of the minimal clinically meaningful difference. This refers to the smallest change in the observed variable that is considered clinically relevant or significant. If the CI includes values within the range of minimal clinically meaningful difference, it may suggest that the observed effect is practically significant.

It is a robust approach to draw meaningful insights from the collected data while considering the practical implications of the findings [28].

### Ethical Considerations

The study has been approved by the University Ethic Committee of Ulm University (77/22). Before study entry, all potential participants are comprehensively informed about the project and a written declaration of consent for participation is obtained. Access to the trial is open to all patients, regardless of their statutory health insurance provider. There are no additional costs or disadvantages for the participating patients. Patients’ participation is voluntary. Participants will be informed verbally and in writing about the nature and scope of the planned procedure before the start of the study. Patients can withdraw their informed consent, including the processing of their data, at any time and without giving reasons. Disclosure takes place in pseudonymized form for the analysis. The names of the participating patients and all other confidential information are subject to medical confidentiality and the provisions of the European General Data Protection Regulation of May 25, 2018. After completion of the analyses, the data are made available on reasonable request in anonymized form in accordance with the institutional regulations and the General Data Protection Regulation. The study results are presented to academic audiences through publication in peer-reviewed journals and presentations at national and international conferences. The results are disseminated in the wider community. If shown to be feasible, the concept will be assessed with regard to its efficacy in reducing postoperative complications.

## Results

This trial started on June 13, 2022, and the first 2 groups of patients have already completed their day clinic sessions. The current day clinic cycle takes place from January to March 2023. Data collection is to be completed by June 2023. As of February 2023, we enrolled 23 participants, 18 of whom were diagnosed with breast cancer, 2 with rectum carcinoma, 1 with esophageal cancer, 1 with stomach cancer, and 1 with pancreatic cancer. We expect the final results to be published by December 2023.

## Discussion

### Overview

This study is meant to prepare patients for surgery taking into consideration physical, nutritional, and psychological aspects. Neoadjuvant treatment is a very sensitive phase in cancer treatment and many patients search for assistance. Up to now this demand often remains unheard. We want to support patients with this comprehensive program, empower them to take control of some of the treatment-associated side effects, and help them prepare for the surgery. Establishing the feasibility of such program is the first step to show the efficacy of comprehensive integrative prehabilitation.

### Principal Results

The main outcome is the adherence to the day clinic program measured via physical attendance at the weekly meetings.

### Limitations

It will not be possible to establish significant data about the efficacy of such an approach as this study is meant to be a feasibility trial. The protocol does not include randomization; hence this is a feasibility study. The importance of the group-based setting may impede recruitment. The restricted cancer types included in the study might hamper the possibility of generalizing the results.

### Conclusions

A PRIME-DC is a promising concept to address many unmet needs of patients undergoing neoadjuvant treatment combining aspects of movement therapy, naturopathic treatment, naturopathic counseling, dietary advice, and other lifestyle modifications like stress management.

## Abbreviations

1-MSTS: 1-minute sit-to-stand test
6-MWD: 6-minute walking distance
EORTC QLQ-C30: European Organisation for Research and Treatment of Cancer Quality of Life Questionnaire C30
FFMQ-D: Five-Facet Mindfulness Questionnaire
HGS: Hand grip strength
MBM: mind-body medicine
PHQ-4: 4-item patient health questionnaire for anxiety and depression
PRIME-DC: prehabilitative in an integrative medicine day clinic
PRO-CTCAE: Common Terminology Criteria for Adverse Events
PSQI: Pittsburgh Sleep Quality Index
QoL: Quality of life
QoR-15: Quality of Recovery-15 score

## Data Availability

The data sets generated and analyzed during this study are available from the corresponding author on reasonable request.

## References

[1] Wynter-Blyth V, Moorthy K (2017). Prehabilitation: preparing patients for surgery. BMJ.

[2] Heger P, Probst P, Wiskemann J, Steindorf K, Diener MK, Mihaljevic AL (2020). A systematic review and meta-analysis of physical exercise prehabilitation in major abdominal surgery (PROSPERO 2017 CRD42017080366). J Gastrointest Surg.

[3] Ebner F, Schulz SVW, de Gregorio A, Volz S, Steinacker JM, Janni W, Otto S (2018). Prehabilitation in gynecological surgery? What do gynecologists know and need to know. Arch Gynecol Obstet.

[4] Gillis C, Gill M, Gramlich L, Culos-Reed SN, Nelson G, Ljungqvist O, Carli F, Fenton T (2021). Patients' perspectives of prehabilitation as an extension of enhanced recovery after surgery protocols. Can J Surg.

[5] Chin CH, Tseng LM, Chao TC, Wang TJ, Wu SF, Liang SY (2021). Self-care as a mediator between symptom-management self-efficacy and quality of life in women with breast cancer. PLoS One.

[6] Britton-Jones CA (2017). Prehabilitation. Br J Hosp Med (Lond).

[7] Mind-body practice. National Cancer Institute.

[8] Paul A, Cramer H, Lauche R, Altner N, Langhorst J, Dobos GJ (2013). An oncology mind-body medicine day care clinic: concept and case presentation. Integr Cancer Ther.

[9] Dobos G, Paul A (2011). Mind-Body-Medizin: die moderne Ordnungstherapie in Theorie und Praxis.

[10] Liu C, Qin M, Zheng X, Chen R, Zhu J (2021). A meta-analysis: intervention effect of mind-body exercise on relieving cancer-related fatigue in breast cancer patients. Evid Based Complement Alternat Med.

[11] Hall DL, Luberto CM, Philpotts LL, Song R, Park ER, Yeh GY (2018). Mind-body interventions for fear of cancer recurrence: a systematic review and meta-analysis. Psychooncology.

[12] Pester BD, Edwards RR, Martel MO, Gilligan CJ, Meints SM (2022). Mind-body approaches for reducing the need for post-operative opioids: evidence and opportunities. J Clin Anesth Intensive Care.

[13] Hanley AW, Gililland J, Erickson J, Pelt C, Peters C, Rojas J, Garland EL (2021). Brief preoperative mind-body therapies for total joint arthroplasty patients: a randomized controlled trial. Pain.

[14] Klafke N, Mahler C, von Hagens C, Uhlmann L, Bentner M, Schneeweiss A, Mueller A, Szecsenyi J, Joos S (2019). The effects of an integrated supportive care intervention on quality of life outcomes in outpatients with breast and gynecologic cancer undergoing chemotherapy: results from a randomized controlled trial. Cancer Med.

[15] Horneber M, Bueschel G, Dennert G, Less D, Ritter E, Zwahlen M (2012). How many cancer patients use complementary and alternative medicine: a systematic review and metaanalysis. Integr Cancer Ther.

[16] (2021). Komplementärmedizin in der Behandlung von onkologischen PatientInnen, Langversion 1.1.

[17] Valentini J, Fröhlich D, Stolz R, Mahler C, Martus P, Klafke N, Horneber M, Frasch J, Kramer K, Bertz H, Grün B, Tomaschko-Ubeländer K, Joos S, CCC-Integrativ study group (2022). Interprofessional evidence-based counselling programme for complementary and integrative healthcare in patients with cancer: study protocol for the controlled implementation study CCC-Integrativ. BMJ Open.

[18] Ghadjar P, Stritter W, von Mackensen I, Mehrhof F, Foucré C, Ehrhardt VH, Beck M, Gebert P, Kalinauskaite G, Luchte JS, Stromberger C, Budach V, Eggert A, Seifert G (2021). External application of liver compresses to reduce fatigue in patients with metastatic cancer undergoing radiation therapy, a randomized clinical trial. Radiat Oncol.

[19] Foucré C, Schulz S, Stritter W, von Mackensen I, Luchte J, Ivaki P, Voss A, Ghadjar P, Seifert G (2022). Randomized pilot trial using external yarrow liver compress applications with metastatic cancer patients suffering from fatigue: evaluation of sympathetic modulation by heart rate variability analysis. Integr Cancer Ther.

[20] Dossett ML, Fricchione GL, Benson H (2020). A new era for mind-body medicine. N Engl J Med.

[21] Fayers P, Bottomley A, EORTC Quality of Life Group, Quality of Life Unit (2002). Quality of life research within the EORTC-the EORTC QLQ-C30. European organisation for research and treatment of cancer. Eur J Cancer.

[22] Basch E, Reeve BB, Mitchell SA, Clauser SB, Minasian LM, Dueck AC, Mendoza TR, Hay J, Atkinson TM, Abernethy AP, Bruner DW, Cleeland CS, Sloan JA, Chilukuri R, Baumgartner P, Denicoff A, St Germain D, O'Mara AM, Chen A, Kelaghan J, Bennett AV, Sit L, Rogak L, Barz A, Paul DB, Schrag D (2014). Development of the National Cancer Institute's patient-reported outcomes version of the common terminology criteria for adverse events (PRO-CTCAE). J Natl Cancer Inst.

[23] Stark PA, Myles PS, Burke JA (2013). Development and psychometric evaluation of a postoperative quality of recovery score: the QoR-15. Anesthesiology.

[24] Kroenke K, Spitzer RL, Williams JBW, Löwe Bernd (2009). An ultra-brief screening scale for anxiety and depression: the PHQ-4. Psychosomatics.

[25] Buysee DJ, Reynolds CF, Monk TH, Berman SR, Kupfer DJ (1989). Pittsburgh sleep quality index. J Clin Psychol Med Settings.

[26] Michalak J, Zarbock G, Drews M, Otto D, Mertens D, Ströhle G, Schwinger M, Dahme B, Heidenreich T (2016). Erfassung von Achtsamkeit mit der deutschen Version des Five Facet Mindfulness Questionnaires (FFMQ-D). Z Gesundheitspsychol.

[27] Clavien PA, Sanabria JR, Strasberg SM (1992). Proposed classification of complications of surgery with examples of utility in cholecystectomy. Surgery.

[28] Lee EC, Whitehead AL, Jacques RM, Julious SA (2014). The statistical interpretation of pilot trials: should significance thresholds be reconsidered?. BMC Med Res Methodol.

